# Characterizing Genotypes and Phenotypes Associated with Dysfunction of Channel-Encoding Genes in a Cohort of Patients with Intellectual Disability

**DOI:** 10.34172/aim.2022.124

**Published:** 2022-12-01

**Authors:** Naeim Ehtesham, Meysam Mosallaei, Maryam Beheshtian, Shahrouz Khoshbakht, Mahsa Fadaee, Raheleh Vazehan, Mehrshid Faraji Zonooz, Parvaneh Karimzadeh, Kimia Kahrizi, Hossein Najmabadi

**Affiliations:** ^1^Genetics Research Center, University of Social Welfare and Rehabilitation Sciences, Tehran, Iran; ^2^Kariminejad – Najmabadi Pathology & Genetics Center, Tehran, Iran; ^3^Department of Pediatric Neurology, School of Medicine, Pediatric Neurology Research Center, Mofid Children’s Hospital, Shahid Beheshti University of Medical Sciences, Tehran, Iran

**Keywords:** Channelopathies, Developmental delay, Genotype, Intellectual disability, Phenotype

## Abstract

**Background:**

Ion channel dysfunction in the brain can lead to impairment of neuronal membranes and generate several neurological diseases, especially neurodevelopmental disorders.

**Methods:**

In this study, we set out to delineate the genotype and phenotype spectrums of 14 Iranian patients from 7 families with intellectual disability (ID) and/or developmental delay (DD) in whom genetic mutations were identified by next-generation sequencing (NGS) in 7 channel-encoding genes: *KCNJ10, KCNQ3, KCNK6, CACNA1C, CACNA1G, SCN8A,* and *GRIN2B*. Moreover, the data of 340 previously fully reported ID and/or DD cases with a mutation in any of these seven genes were combined with our patients to clarify the genotype and phenotype spectrum in this group.

**Results:**

In total, the most common phenotypes in 354 cases with ID/DD in whom mutation in any of these 7 channel-encoding genes was identified were as follows: ID (77.4%), seizure (69.8%), DD (59.8%), behavioral abnormality (29.9%), hypotonia (21.7%), speech disorder (21.5%), gait disturbance (20.9%), and ataxia (20.3%). Electroencephalography abnormality (33.9%) was the major brain imaging abnormality.

**Conclusion:**

The results of this study broaden the molecular spectrum of channel pathogenic variants associated with different clinical presentations in individuals with ID and/or DD.

## Introduction

 Dysfunction of ion channels, both ligand- and voltage-gated, generate a group of disorders called channelopathies. Deficiency of ion channels results in gain of channel function (i.e. hyper-) or loss of channel function (i.e. hypo-) excitability of the affected tissue.^1^ Transient and recurrent derangement of membrane excitability in the brain probably leads to paroxysmal or episodic symptoms, especially seizures and ultimately developmental delay (DD).^2^ In the central and peripheral nervous system, the existing ion channel proteins are involved in cell migration, neuronal differentiation, and axonal integrity via modulation of multiple signaling pathways.^3,4^ The decreasing cost of next-generation sequencing (NGS) has led to the identification of pathogenic mutations in well-phenotyped patient cohorts, especially neuropsychiatric and neurodevelopmental disorders.^5,6^ According to Gene Ontology (GO) terms, about 700 genes bear a channel function activity (http://amigo.geneontology.org/amigo/term/GO:0015267). The association between different types of channelopathies such as calcium-,^7^ potassium-,^8^ sodium-,^9^ and ligand-gated^10^ channels with intellectual disability (ID) has been well-established. Up to now, a constellation of phenotypic manifestations and genotype frequencies have been reported for channel-associated ID and/or DD. Hence, in this study, we aimed to present the array of phenotypic symptoms and molecular spectrum of 14 Iranian patients from 7 families with ID and/or DD in whom a genetic variant in one channel-encoding gene has been previously identified. They were selected from a large Iranian cohort of families with ID and/or DD. Likewise, we combined the data of our cases with previously published papers in which ID/DD patients with a mutation in one channel-encoding were recruited. The results of this study widen the association between clinical phenotype and channel pathogenic variants in individuals with ID and/or DD.

## Materials and Methods

 As a case-series study, we selected 14 ID and/or DD patients from 7 families of Iranian descent in whom the segregation of disease-causing variants in channel-encoding genes has been confirmed. For recruiting these patients, a large Iranian cohort was considered. The cohort consisted of 540 mostly consanguineous ID and/or DD unions with ≥ 2 affected individuals, and 100 single-patient families who were visited in the Genetics Research Center (GRC) at the University of Social Welfare and Rehabilitation Sciences (USWR) between 2007 and 2018. All the patients underwent full clinical examination. Patients with known ID diseases such as Down syndrome, fragile X syndrome, cerebral palsy, and phenylketonuria were excluded based on phenotype and/or metabolic screening. Also, autism patients with ID and patients with ID-associated degenerative disorders comprising hereditary spastic paraplegia, and spinocerebellar ataxia were included in our study. Utilizing the salting-out method, DNA was elicited from 2 cc of EDTA anti-coagulated venous blood samples of patients and their related family members.^11^ Following the quantification of extracted DNA through the exploitation of the NanoDrop 2000 (Thermo Scientiﬁc) device, nearly 2 µg of DNA was used for NGS. All the samples were subjected to whole-exome sequencing (WES) employing different versions of the Agilent SureSelect kit for the enrichment of the exome target. The American College of Medical Genetics/Association of Molecular Pathology (ACMG/AMP) guidelines were leveraged to interpret the pathogenicity of identified variants.^12^ Our methodology for molecular diagnosis was previously described in detail.^13-15^ A parent or a legal guardian of all participants in this study provided written informed consent and the Ethics Committee of the USWR approved the study. Ultimately, the clinical phenotypes and molecular lesions of other deeply phenotyped published cases were gathered up by conducting a comprehensive search in PubMed, Scopus, the ISI Web of Science, and Google Scholar up to June 2021 to delineate the range of channel-associated phenotypes and mutations in patients presenting with ID and/or DD. To avoid non-conformity in the usage of terms for clinical presentations, Human Phenotype Ontology (HPO) was harnessed (https://hpo.jax.org/).

## Results

 Out of 14 patients from 7 independent families, 10 (71%) subjects were males and 4 (29%) were females. All affected individuals were born to consanguineous parents and were the products of uneventful pregnancies and deliveries. Concerning ID as the cardinal symptom of all the patients, the severity of ID in 9 (64%) and 5 (36%) individuals was severe and moderate, respectively. The data about the genotype and phenotype of selected patients are summarized in Table 1 and Table 2, respectively. The pedigree of each family is depicted in Figure S1 (See Supplementary file 1).

**Table 1:** Demographic Data and Genetic Variants of 7 Iranian Families with a Mutation in 7 Channel-encoding Genes

**Family Number/ Ethnicity**	**Gene Symbol**	**Number of Affected/ Sex**	**Parental Relationship**	**Variation**	**Mutation Type**	**Zygosity/ Inheritance**	**Protein Domain**
1/Persian	*KCNJ10*	2/2 M	First cousin	Chr1: g.160041778G > C; NM_002241.4: c.755C > G (p. Pro252Arg)	Missense	Hom /AR	IRK_C
2/ Persian	*KCNQ3*	1/F	First cousin	Chr8: g.132180246G > A; NM_004519.3: c.688C > T (p. Arg230Cys)	Missense	Het /AD	Fourth transmembrane of ion transport domain
3/ Persian	*KCNK6*	2/ 1F, 1 M	First cousin	Chr19: g.19:38817538A > C; NM_004823: c.628A > C (p. Ile210Leu)	Missense	Hom /AR	Second transmembrane of ion transport domain 2
4/ Persian	*CACNA1C*	3/ 1F, 2 M	First cousin	Chr12: g.12:2622142C > A; NM_199460: c.1382C > A (p.Pro 461His )	Missense	Hom /AR	—
5/ Turkmens	*CACNA1G*	3/ 3 M	First cousin	chr17:46035496-46035625del130; NM_018896: p.Ser1346fs	Frameshift	Hom /AR	Third transmembrane
6/ Persian	*SCN8A*	2/ 1F, 1 M	Third Cousin	Chr12: g.12:52115484G > A; NM_014191: c.1790G > A (p. Arg597His)	Missense	Hom /AR	—
7/ Persian	*GRIN2B*	1/ M	First cousin	Chr12: g.13724864C > T; NM_000834.3: c.2045G > A (p. Arg682His)	Missense	Het/ De novo	PBPe

M, Male; F, Female; AR, Autosomal recessive; AD, Autosomal dominant; Hom, Homozygous; Het, Heterozygous; IRK, Inward-rectifier potassium channels; PBPe, Eukaryotic homologues of bacterial periplasmic substrate binding proteins.

**Table 2:** Clinical and Radiological Findings in 14 Patients of Our Cohort with Disease-associated Variants in 7 Channel-Encoding Genes

**Family **	**Gene Symbol**	**Diagnosis**	**Clinical Findings**
1	*KCNJ10*	Syndromic ID	DD (HP:0001263); ID (HP:0001249), severe; Seizure (HP: HP:0001250); Facial dysmorphism (HP:0001999) including long face, open and large mouth; Behavioral abnormality (HP:0000708) including Drooling, and easily overstimulated; Hypotonia (HP:0001252), mild
2	*KCNQ3*	GDD	DD (HP:0001263); ID (HP:0001249), severe, Refractory seizures (HP:0001250); Behavioral abnormality (HP:0000708) including autistic behavior (HP:0000729); Strabismus (HP:0000486); No Facial dysmorphism (HP:0001999), Normal MRI
3	*KCNK6*	Syndromic ID	ID (HP:0001249), severe; Microcephaly (HP:0000252) (OFC: –2.5SD to –5SD); Hypotonia (HP:0001252); Psychomotor delay (HP:0001263); Speech disorder (HP:0002167) including slurred speech (HP:0001350); Inability to walk (HP:0002540); Poor suck (HP:0002033); Short stature (HP:0004322); No seizures (HP:0001250); No Facial dysmorphism (HP:0001999)
4	*CACNA1C*	non-syndromic ID	ID (HP:0001249), moderate; No seizures (HP:0001250); No cardiac involvement; No Microcephaly (HP:0000252)
5	*CACNA1G*	non-syndromic ID	ID (HP:0001249), moderate to severe; Psychomotor delay (HP:0001263), severe; Cataract (HP:0000518); Ataxia (HP: 0001251); Hypertonia (HP:0001276); facial dysmorphism (HP:0001999)
6	*SCN8A*	syndromic ID	ID (HP:0001249), moderate; Microcephaly (HP:0000252) (OFC:-5.4SD to -6.1SD); Short stature (HP:0004322) (-3.7SD to -4.1 SD); No seizures ( HP:0001250)
7	*GRIN2B*	syndromic ID	ID (HP:0001249), moderate to severe; Behavioral abnormality (HP:0000708) including obsessive behavior but autistic behavior (HP:0000729); Facial dysmorphism (HP:0001999) including narrow face (HP:0000275) and maxillary hyperplasia (HP:0430028); No seizure (HP:0001250); No hypertonia (HP:0001276); No microcephaly (HP:0000252)

Family ID, Family identifier; ID, Intellectual disability; DD, Developmental delay; MRI, Magnetic resonance imaging; SD, Standard deviation; OFC, occipito-frontal head circumference; HP, The Human Phenotype Ontology (HPO) identifier.

 The identified missense variant in *KCNJ10* (c.755C > G; p.Pro252Arg) has not been previously documented in the literature. This variant lies on the cytoplasmic C-terminal pore domain and was inherited in a homozygous manner. The mutations of *KCNJ10* underlie SeSAME/EAST syndrome, in which ID is not an obligate symptom of the disease.^16^ However, our patients demonstrated severe ID. Akin to other previously reported cases (Table S1.1, see Supplementary file 2), patients of family 1 experienced onset of seizures at infancy (before 6 months).

 A WES analysis revealed a mutation in the hotspot site of *KCNQ3* (230th codon) in family 2. According to Table S1.2, out of 31 patients with mutations in this gene, 17 (54.8%) harbored mutation at this place (p.R230H; p.R230S; p.R230C; and p.R230L). The same mutation was previously indicated in patients with autism and developmental disability,^17^ global developmental delay (GDD),^18^ and cerebral visual impairment with ID.^19^ The affected female had GDD with these milestones characteristics: smiled at 3 months, rolled over at 10 months, sat at 11 months without support, at 18 months stood without support, the first word at 1.5 years of age, head up at 3 months, crawled at 16 months, and walked at 20 months with frequent falling. Currently, at the age of 3 years, she knows only 30-35 words. She also had a history of hospitalization due to high fever at the age of 18 months.

 Our previous study, for the first time, revealed that mutation in *KCNK6* could be the novel underlying cause of autosomal recessive ID (ARID).^14^ To the best of authors’ knowledge, there is no other report about the association between the mutation in this gene with ID and/or DD. Two sibs of family 3 had psychomotor delay including head control at age 10 and 12 months, respectively, sitting at age 16 months, and starting to walk at age 5 years. The elder patient did not have bowel or bladder control. She did not have the ability to communicate, to know colors and her family names, nor to follow simple commands. The proband (III: 2, Figure S1) had very little interest in his environment and very little interest in playing. Both had normal vision and at 14 and 6 years, their heights were 132 (< 3 percentile) and 113 cm (50 percentile), respectively.

 Three affected subjects of family 4 inherited the pathogenic missense variant of *CACNA1C *in a homozygous manner and represented non-syndromic ID.^14^ This is in contrast with previous studies that reported heterozygous *de novo* mutations in this gene can give rise to Timothy syndrome (Table S1.3).^20,21^ The only frameshift mutation in our cases belonged to family 5 in which a 130 bp deletion was found in another voltage-gated calcium channel named *CACNA1G*.^13^ This deletion involves removing part of the 3´ end of exon 21 (NM_018896) and the flanking intron, and is predicted to cause deletion of the extracellular domain between S3 and S4 helices and also some part of the S4 helix which subsequently abolishes its function.

 In family 6, the disease-causing variant was found in *SCN8A*. In contrast with previous reports in which *SCN8A*- associated ID and/or DD phenotypes were inherited as heterozygous (Table S1.4), homozygous inheritance was noted in this family. In other words, the phenotypes of our cases match with previously reported patients, but a different inheritance pattern runs in our investigated family.^14^

 Sequencing in a family with sporadic ID revealed a dominant *de novo* mutation in *GRIN2B*, the only ligand-gated ion channel in our cohort. The association of variants in *GRIN2B* with neurological, psychiatric, and neurodevelopmental disorders, especially ID (0.5–1% in patients with ID), has been indicated (Table S1.5).^22^

 The clinical findings of our and other ID and/or DD cases are provided in Table S2 separately for each gene and combined on the first sheet (Table S2.1, Supplementary file 3). Also, in Table 3, the molecular characteristics and the most common phenotypes of 352 patients (including unavailable data) with disease-causing variants in 6 channel-encoding genes are presented. The data of the *KCNK6 *gene was not incorporated in Table 3 because the only reported ID/DD family with a mutation in this gene was family 7 in our study (Table S1.6). Collectively, the most common phenotypes in 354 cases with ID/DD in whom mutation in any of these 7 channel-encoding genes was identified were as follows: ID (77.4%), seizure (69.8%), DD (59.8%), behavioral abnormality (29.9%), hypotonia (21.7%), speech disorder (21.5%), gait disturbance (20.9%), and ataxia (20.3%) (Table S2.1). Electroencephalography (EEG) abnormality (33.9%) was the major brain imaging abnormality. The detailed phenotypic and molecular spectrum of each gene is represented in the discussion.

**Table 3:** Clinical and Molecular Characteristics of 352 Patients (Including Unavailable Data) with Disease-associated Variants in 6 Channel-encoding Genes in Percentage

**Molecular and Clinical Characteristics**	***KCNJ10 *** **(N:54)**	***KCNQ3 *** **(N:31)**	***CACNA1C *** **(N:16)**	***CACNA1G *** **(N:17)**	***SCN8A *** **(N:135)**	***GRIN2B *** **(N:99)**
**Frequency of Variant Types**						
Missense	47 (87 %)	27 (87%)	14 (87.5%)	14 (82.3%)	132 (95.6%)	84(84.8%)
Frameshift	5 (9.2%)	4 (13%)	0 (0%)	3 (17.6%)	2 (1.4%)	5 (5%)
Nonsense	2 (3.7%)	0 (0%)	2 (12.5%)	0 (0%)	0 (0%)	4 (4%)
Splice site	0 (0%)	0 (0%)	0 (0%)	0 (0%)	4 (2.9%)	4 (4%)
Indel	0 (0%)	0 (0%)	0 (0%)	0 (0%)	0 (0%)	1 (1.01%)
GOF/LOF/NA of unique mutations (#)	2 (3.8%)/40 (76.2%)/10 (19.2%) (#52)	8 (32%)/7 (28%)/10 (40%) (#25)	2 (22.2%) /1 (11.1%) /6 (66.6%) (#9)	7 (87.5%)/1 (14.3%)/1 (14.3%) (#8)	25 (19.5%)/13 (10.1%)/90 (70.3%)(#128)	8 (19.5%)/0 (0%)/81 (91%)(#89)
**Abnormalities of Head and Neck**						
Nystagmus (HP: 0000639)	7P/10N/37NA (12.9%)	1P/1N/29NA (3.2%)	0P/0N/16NA (0%)	2P/7N/8NA (11.7%)	3P/0N/132NA (2.2%)	1P/0N/98NA (1%)
Strabismus (HP:0000486)	0P/0N/54NA (0%)	14P/0N/17NA (45.1%)	0P/1N/15NA (0%)	7P/0N/10NA (41.2%)	2P/0N/133NA (1.5%)	1P/0N/98NA (1%)
Facial dysmorphism^a^(HP:0001999)	9P/1N/44NA (16.6%)	5P/1N/25NA (16.1%)	9P/1N/6NA (56.2%)	12P/0N/5NA (70.6%)	4P/4N/127NA (2.9%)	5P/6N/88NA (5%)
Microcephaly (HP:0000252)	2P/0N/52NA (3.7%)	3P/2N/26NA (9.7%)	0P/3N/13NA (0%)	3P/6N/8NA (17.6%)	9P/0N/126NA (6.6%)	12P/5N/82NA (12.1%)
**Abnormality of Growth**						
Low body weight (HP:0004325)	4P/1N/49NA (7.4%)	4P/2N/25NA (12.9%)	1P/0N/15NA (6.2%)	0P/0N/17NA (0%)	0P/0N/135NA (0%)	1P/0N/98NA (1%)
Short stature (HP:0004322)	4P/0N/50NA (7.4%)	4P/2N/25NA (12.9%)	1P/0N/15NA (6.2%)	0P/0N/17NA (0%)	3P/0N/132NA (2.2%)	0P/0N/99NA (0%)
**Neurological Features**						
Developmental delay (HP: 0001263)	46P/1N/7NA (85.2%)	27P/1N/3NA (87%)	8P/1N/7NA (50%)	16P/0N/1NA (94.1%)	81P/3N/51NA (60%)	32P/0N/67NA (32.3%)
Intellectual disability (HP:0001249)	19P/13N/22NA (35.2%)	29P/1N/2NA (95.5 %)	8P/0N/8NA (50%)	16P/0N/1NA (94.1%)	106P/1N/28NA (78.5%)	94P/0N/5NA (94.9%)
Seizure (HP:0001250)	54P/0N/0NA (100%)	17P/11N/3NA (54.8%)	7P/6N/3NA (43.7%)	7P/7N/3NA (41.2%)	127P/5N/3NA (94%)	35P/7N/57NA (35.3%)
Ataxia (HP: 0001251)	45P/1N/8NA (83.3%)	5P/2N/24NA (16.1%)	0P/0N/16NA (0%)	8P/0N/9NA (47%)	14P/0N/121NA (10.4%)	0P/0N/99NA (0%)
Incoordination^b^ (HP: 0002311)	7P/0N/47NA (12.9%)	1P/2N/28NA (3.2%)	0P/0N/16NA (0%)	0P/0N/17NA (0%)	3P/0N/132NA (2.2%)	0P/0N/99NA (0%)
Hypertonia (HP:0001276)	8P/0N/46NA (14.8%)	3P/0N/28NA (9.7%)	0P/0N/16NA (0%)	12P/4N/1NA (70.6%)	8P/0N/127NA (5.9%)	1P/0N/98NA (1%)
Hyperreflexia^c^ (HP:0001347)	17P/5N/32NA (31.5%)	1P/2N/28NA (3.2%)	1P/0N/15NA (6.2%)	5P/4N/8NA (29.4%)	4P/0N/131NA (2.9%)	0P/0N/99NA (0%)
Speech disorder (HP: 0002167)	23P/3N/28NA (42.5%)	22P/0N/9NA (70.9%)	1P/0N/15NA (6.2%)	6P/0N/11NA (35.3%)	19P/0N/116NA (14%)	3P/0N/96NA (3%)
Gait disturbance^d^ (HP:0001288)	33P/2N/19NA (94.3%)	7P/1N/23NA (22.6 %)	0P/0N/16NA (0%)	12P/2N/3NA (70.6%)	18P/0N/117NA (13.3%)	2P/0N/97NA (2%)
Hypotonia (HP:0001252)	10P/0N/44NA (18.5%)	11P/2N/18NA (35.5%)	2P/1N/13NA (12.5%)	10P/1N/6NA (58.8%)	37P/3N/95NA (27.4%)	6P/0N/93NA (6%)
Involuntary movements^e^ (HP:0004305)	23P/2N/29NA (42.6%)	0P/2N/29NA (0%)	0P/0N/16NA (0%)	0P/0N/17NA (0%)	29P/2N/104NA (21.5%)	1P/0N/98NA (1%)
**Skeletal Abnormalities**						
Abnormal foot morphology^f^ (HP:0001760)	3P/7N/44NA (5.5%)	2P/0N/29NA (6.4%)	3P/0N/13NA (18.7%)	1P/0N/16NA (5.9%)	0P/0N/135NA (0%)	0P/0N/99NA (0%)
Abnormal digit morphology^g^ (HP:0011297)	0P/0N/54NA (0%)	0P/0N/31NA (0%)	7P/5N/4NA (43.7%)	9P/0N/8NA (52.9%)	1/0N/134NA (0.7%)	0P/0N/99NA (0%)
Flexion contracture (HP:0001371)	1P/0N/53NA (1.8%)	0P/0N/31NA (0%)	5P/0N/11NA (31.2%)	1P/0N/16NA (5.9%)	0P/0N/135NA (0%)	0P/0N/99NA (0%)
Behavioral abnormalities						
Autistic behavior^h^ (HP:0000729)	10P/10N/34NA (18.5%)	17P/3N/11NA (54.8%)	0P/1N/15NA (0%)	5P/6N/66NA (29.4%)	12P/23N/100NA (8.8%)	25P/5N/69NA (25.2%)
Sleep disturbance (HP:0002360)	3P/17N/34NA (5.5%)	1P/19N/11NA (3.2%)	3P/1N/15NA (18.7%)	0P/0N/17NA (0%)	1P/34N/100NA (0.7%)	2P/28N/69NA (2 %)
**Brian Imaging Findings**						
EEG abnormality (HP:0002353)	20P/9N/25NA (37%)	15P/5N/11NA (48.4%)	2P/1N/13NA (12.5%)	9P/3N/5NA (52.9%)	70P/8N/57NA (51.8%)	4P/1N/94NA (4%)
Cerebellar atrophy (HP:0001272)	5P/5N/43NA (9.2%)	0P/0N/31NA (0%)	0P/0N/16NA (0%)	6P/0N/11NA (35.3%)	15P/0N/120NA (11.1%)	0P/0N/99NA (0%)
Other MRI findings^l^	20P/16N/18NA (37%)	6P/9N/16NA (19.3%)	1P/0N/15NA (6.2%)	2P/0N/15NA (11.7%)	6P/6N/123NA (4.4%)	0P/0N/99NA (0%)
Other features						
Abdominal symptom^m^ (HP:0011458)	3P/0N/51NA (5.5%)	1P/1N/29NA (3.2%)	2P/0N/14NA (12.5%)	1P/0N/16NA (5.9%)	6P/2N/127NA (4.4%)	3P/0N/96NA (3%)
Abnormality of the respiratory system^n^ (HP:0002086)	0P/0N/54NA (0%)	1P/0N/30NA (3.2%)	6P/0N/10NA (37.5%)	0P/0N/17NA (0%)	8P/0N/127NA (5.9%)	0P/0N/99NA (0%)

N, negative; P, positive; NA, not available; GOF, gain of function; LOF, Loss of function; EEG, Electroencephalography; MRI, Magnetic resonance imaging. ^a^Including Retrognathia (HP,0000278), Low-set ears (HP,0000369), Short palpebral fissure (HP,0012745), Abnormal facial shape (HP,0001999) comprising Long face (HP,0000276), Wide mouth (HP,0000154), Thick lower lip vermilion (HP,0000179), Hypertelorism (HP,0000316), Synophrys (HP,0000664), and…. ^b^Including Clumsiness (HP,0002312). ^c^Including Brisk reflexes (HP,0001348), Clonus (HP,0002169), and Jaw hyperreflexia HP,0033683. ^d^Including Inability to walk (HP,0002540), and Broad-based gait (HP,0002136). ^e^Including Tremor (HP,0001337), Myoclonus HP,0001336, Tics HP,0100033, Paroxysmal dyskinesia HP,0007166, Athetosis HP,0002305. ^f^Including Talipes equinovarus (HP,0001762), Pes planus (HP,0001763), and Pes cavus (HP,0001761). ^g^Including Syndactyly (HP,0001159), Camptodactyly (HP,0012385), and Clinodactyly (HP,0030084), and Broad thumb (HP,0011304). ^h^Including Stereotypy (HP,0000733), Impaired social interactions (HP,0000735), Poor eye contact (HP,0000817). ^l^Including Thin corpus callosum (HP,0033725), Anomaly of the basal ganglia (HP,0002134), Hypoplasia of the pons (HP,0012110), Abnormality of the dentate nucleus (HP,0100321), Perivascular spaces (HP,0012520), and Periventricular white matter abnormalities (HP,0002518). ^m^Including Constipation (HP,0002019), Feeding difficulties (HP,0011968), Vomiting (HP,0002013), and Bloody diarrhea (HP,0025085). ^n^Cyanosis HP:0000961, Stridor HP:0010307, Laryngomalacia HP:0001601, Dyspnea HP:0002094, Bronchitis HP:0012387, Apnea HP:0002104, Asthma HP:0002099.

## Discussion

 Given the pivotal role of all channel isoforms in the generation and conduction of action potentials in the brain, their impairment can be associated with the development of a wide range of disorders termed neurological channelopathies.^23^ Here, we discuss the genotype and phenotype of combined data of our and other published cases in the spectrum of channel-related diseases for each gene separately.

###  Voltage-Gated Potassium Channels

 Potassium channels that are endowed with diverse gating properties, participate in the modulation of membrane resistance and action potentials, spike frequency, and potential of resting membrane.^24^ Dysfunction of potassium channels is associated with several neurological disorders especially ID and epilepsy. However, the contribution of potassium channelopathies to ID is not hitherto clear.^8^ SeSAME/EAST syndrome with autosomal-recessive inheritance pattern is a multi-systemic neuropsychiatric disease that emanates from compound heterozygous or homozygous mutations in *KCNJ10*, encoding Kir4.1, an ATP-sensitive inwardly rectifying potassium channel.^25^ Out of 54 patients with *KCNJ10* mutation, 47 patients (87%) had missense, 5 (9.2%) had frame-shift, and 2 (3.7%) had a nonsense mutation (Table 3). The c.193C > T (p.R65C) was the most recurrent mutation (17 out of 54 31.5%; Table S1.1) in *KCNJ10*, manifesting the presence of a hotspot for mutations. Although this most frequently reported mutation is juxtaposed near the first transmembrane domain at the N-terminal cytoplasmic side, a large number of variants are seen intensively at cytoplasmic C-terminal pore domain.^26^ In terms of zygosity, most variants were inherited in a homozygous state (42 of 54: 77.7%; Table S1.1). Patients with *KCNJ10* mutation were mostly male (34 out of 53: 64%). The average age of the affected individuals at the last examination and onset was 9.6 years and 4.5 months, respectively. Out of 48 cases whose parental relationship was reported, 29 (60%) had related parents. The most prevalent symptoms of the affected individuals who harbored *KCNJ10* mutation were seizure (100%), DD (85.2%), ataxia (83.3%), sensorineural hearing loss (68.5%), abnormal blood ion concentration including hypokalemia, hypomagnesemia, hyponatremia, and hypochloremia (66.6%), gait disturbance (61.1%), urinary electrolyte imbalance (52.2%), involuntary movements (42.6%), speech disorder subsuming dysarthria and absent speech (42.5%), ID (35.2%), hyperreflexia (31.5%), proximal tubulopathy (29.6%), behavioral abnormality (27.7%), and pyramidal signs (20.7%) (Table S2.2). Major characteristic brain imaging findings entailed EEG abnormality (37%) followed by thin corpus callosum (29.6%) (Table S2.1). Obviously, infantile-onset of seizures was consistently noticed in all the patients with *KCNJ10* mutation. In patients with *KCNJ10* mutation, the clinical presentation can be variable, even within a sibship. This indicates the association between *KCNJ10* mutation and a wide range of phenotypes. In this context, patients with the same mutation in *KCNJ10* (p.T290A) showed different degrees of sensorineural hearing loss ranging from mild to severe.^26^ The majority of reported mutations in *KCNJ10* lead toward loss of function (76.2%; Table 3). This mechanism results in mild ID,^27^ whereas, depending on the variant, gain of function mutations are associated with mild to severe ID.^28^ About 40% of the investigated patients, including ours, failed to manifest renal electrolyte deficit. There could be two possible scenarios for this discrepancy. First, possibly, given *KCNJ10* mutations can affect brain functions independently of other organs in the body on account of the greater sensitivity of neurons to deregulation of potassium homeostasis than that of the basolateral membrane of nephron. Second, regarding that channel activity is contingent greatly upon the formation of tetramers with other Kir entities (Kir5.1), the similar *KCNJ10* variants could affect the kidney and CNS differently. Regardless of these two possibilities, the normal serum electrolyte levels in some studies could be attributed not to the manifestation of ion concentration abnormalities in patients younger than 3 years.^29^ Lack of recognition of ID in 65% of the investigated cases could be because that ataxia and hearing loss make the cognitive assessment of the patients harder.^16^ However, it has been posited that ID is not an indispensable result of *KCNJ10* mutations,^30^ mirrored in our results (35%; Table S2.1).

*KCNQ3* (potassium voltage-gated channel subfamily Q member 3) encodes the Kv7.3 neuronal voltage‐gated potassium (K + ) channel subunit.^31^ Regarding the Gene Review (https://www.ncbi.nlm.nih.gov/books/NBK201978/, accessed on May 2021), the KCNQ3-related disorder comprises three kinds of clinical presentations: benign familial neonatal epilepsy, benign familial infantile epilepsy, and *KCNQ3*-related DD. In this study, we only considered the third group of patients. Out of 31 patients with *KCNQ3* mutation, 27 patients (87%) had missense, and 4 (13%) had frame-shift mutations (Table 3). *KCNQ3* variants were mostly inherited in a heterozygous manner (24 of 31; 77.4%) of which 18 arose *de novo* (75%) (Table S1.2). Among patients with *KCNQ3* mutation, the predominant gender was male (18 of 31; 58%). The average age of the affected individuals at the last examination was 6.5 years. The most prevalent symptoms of affected individuals who harbored *KCNQ3* mutation were ID (95.5%), DD (87%), speech disorder (70.9%), behavioral abnormality (58%), seizures (54.8%), strabismus (45.1%), hypotonia (35.5%), and gait disturbance (22.6%) (Table S2.3). The major abnormal brain imaging finding was EEG abnormality (48.4%). In some exceptional cases of familial transmission of the *KCNQ3* pathogenic variant, the disease severity was different between the patient and his son.^32^ Intriguingly, both gain and loss of function mutations of *KCNQ3 *cause moderate and severe ID^17,33^; however, the reason for this phenomenon remains to be deciphered.

###  Voltage-Gated Calcium Channels

 Calcium channels have a major role in the electrical excitability of neurons; therefore, mutations in genes coding for calcium channels are most probably associated with neurodegenerative and neurodevelopmental disorders.^34^ *CACNA1C* encodes Cav1.2 which is an L-type calcium voltage-gated channel. Pathogenic heterozygous variants in this gene lean toward an extremely rare disorder named Timothy syndrome (OMIM: 601005), which is characterized by multisystem abnormalities consisting of facial dysmorphisms, cardiac, and limb anomalies, and neurologic features.^35^ Out of 16 patients with *CACNA1C* mutation, 14 patients (87%) had missense, and 2 (13%) had splice site mutations (Table 3). Two recurrent mutations were p.Gly406Arg in the alternatively spliced exon 8A and p.Gly402Ser in exon 8. Except for our patient, the pathogenic variant in all other patients was inherited in a heterozygous status and had occurred *de novo* (Table S1.3). The number of males with *CACNA1C* mutation was higher than females (9 of 15; 60%). The average age of the affected individuals at the last examination was 3 years. Most of the patients with *CACNA1C* mutation were born to non-consanguineous marriage (5 out of 7; 71%). The most prevalent symptoms of affected individuals who harbored *CACNA1C* mutation were cardiac anomaly (68.7%) comprising tetralogy of Fallot, left ventricular noncompaction, ventricular septal defect, patent ductus arteriosus, and endocardial fibroelastosis, facial dysmorphism (56.2%), DD (50%), ID (50%), abnormal digit morphology such as syndactyly (43.7%), seizure (43.7%), abnormality of the respiratory system (37.5%), flexion contracture (31.2%), and behavioral abnormality (25%). Abnormal electrocardiography was the predominant imaging abnormality (50%) (Table S2.4). Variable expressivity of symptoms has been seen within a family with *CACNA1C* mutation.^36^ The most common mutation (p.Gly406Arg) is situated at transmembrane segment S6 of Domain I, mirroring a hotspot.^37^ Moreover, it is assumed that the predominant effect of mutations on Cav1.2 function is the loss of voltage-dependent inactivation,^38^ however, our result does not tally with this assumption (Table 3: 11% loss of function). Of particular note, in contrast with other cases, the patient of this study had non-syndromic ID.

 The pore-forming subunit of the low-voltage-activated (T-type) Cav3.1 channel is codified by *CACNA1G*. The expression of *CACNA1G *is distributed in different parts of the CNS, especially in Purkinje neurons and the deep nuclei of the cerebellum.^39^ Except for family 5 of this study, the pathogenic variants in all other patients were inherited in a heterozygous status and had occurred *de novo* (Table S1.7). *De novo *gain-of-function pathogenic variants in *CACNA1G* result in an ultra-rare autosomal dominant syndrome that is related to various forms of cerebellar ataxia and neurological comorbidities.^40,41^ All patients with this early-onset syndrome share common features entailing GDD, axial hypotonia, and dysmorphic features, and exhibit cerebellar atrophy and/or hypoplasia on neuroimaging.^42^ The most recurrent reported mutation was c.2881G > A (p.Ala961Thr) (8 out of 17 patients; 47%). This hotspot variant is located within the transmembrane intracellular segment S6 of domains II and III, respectively, and affects as a gain-of-function mutation.^43^ Out of 17 patients with *CACNA1G* mutation, 14 patients (82.3%) had missense, and 3 (17.6%) had frame-shift (Table 3). The affected females with *CACNA1G* mutation were more prevalent than males (66.6%). The average age of the affected individuals at the last examination and onset was 8.7 years and 3.7 months, respectively. The most prevalent symptoms of affected individuals who harbored *CACNA1G* mutation were DD (94.1%), ID (94.1%), gait disturbance (70.6%), hypertonia (70.6%), facial dysmorphism (70.6%), behavioral abnormality (64.7%), hypotonia (58.8%), spastic paraplegia (52.9%), abnormal digit morphology (52.9%), ataxia (47%), seizure (41.2%), strabismus (41.2%), and speech disorder (35.3). The most prominent neuroimaging findings were EEG abnormality (52.9%), and cerebellar atrophy (35.3%) (Table S2.5).

###  Voltage-Gated Sodium Channels

 Voltage-gated sodium channels are implicated in excitability of electrically excitable cells like neurons. Therefore, mutations in sodium channel-encoding genes are associated with concomitant brain and other-organ phenotypes. Severe early-onset epileptic encephalopathies emanate from brain sodium channelopathies.^9^ *SCN8A*, which is extensively expressed in the brain, encodes the pore-forming voltage-gated sodium channel Nav1.6 alpha subunit. SCN8A-developmental and epileptic encephalopathy (DEE) or early infantile epileptic encephalopathy type 13 (EIEE13) (OMIM #614558) is a severe rare autosomal dominant disorder characterized by early-onset intractable seizures, moderate to severe ID, cortical vision impairment, lack of language, motor disorders, inability to walk, and elevated risk of sudden unexpected death.^44^ Variable phenotypes emanate from different *SCN8A* mutations. In this regard, loss of function mutations that lower firing predominantly result in ID, myoclonus, autism spectrum disorder, and ataxia with or without epilepsy, while gain of function mutations with increased neuronal firing usually lead to severe developmental epileptic encephalopathies.^45,46^ In *EIEE13*, most of the reported mutations localize on highly conserved segments of the protein i.e. transmembrane segments, inactivation gate, and proximal 2/3 of the C-terminal.^47^ Out of 135 patients with *SCN8A* mutation, 132 patients (95.6%) had missense, 2 (1.4%) had frame-shift, and 4 (2.9%) had splice site mutation (Table 3). Position 1872 (CGG; an arginine) at SCN8A protein is a mutational hot spot because 18 patients with different types of recurrent amino acid substitutions mutation at this site have been reported so far. However, an overt association between a variant at Arg1872 and the severity of the clinical symptoms does not exist.^44^ Another CpG dinucleotide residue in which amino acid substitutions occur is arginine codon 1617 (CGA), which has been identified in more than ten independent patients (Table S1.4). Irrespective of two cases with compound heterozygous and homozygous variants, all other *SCN8A* variants were inherited in a heterozygous status (133 of 135; 98.5%; Table S1.4), mostly occurring as *de novo* (126 of 133; 94.7%) and others inherited from an unaffected somatic mosaic parent. Out of 107 patients in whom the sex was determined, 60 were female (56%). The average age of the affected individuals at the last examination and onset was 6.6 years and 4.4 months, respectively. The most prevalent symptoms of affected individuals who harbored *SCN8A* mutation were seizure (94%), ID (78.5%), DD (60%), hypotonia (27.4%), involuntary movements (21.5%), and behavioral abnormality (20.7%). EEG abnormality (51.8%), and cerebellar atrophy (11.1%) were the most prominent neuroimaging findings (Table S2.6).

## Ligand-Gated Ion Channels

 GRIN genes such as *GRIN2B*/*GluN2B *encode subunits of a glutamate-gated ion channel named N-methyl-D-aspartate receptors (NMDARs) that crucially take part in neuronal development, various cognition-related functions encircling learning and memory, and synaptic plasticity by the mediation of permeability of Ca^2 +^ component of excitatory postsynaptic transmission in CNS.^48^ Out of 99 patients with *GRIN2B* mutation, 84 patients (84.8%) had missense, 5 (5%) had frame-shift, 4 had nonsense (4%), 4 had splice site (4%), and one (1%) had indel mutation (Table 3). All the reported variants were in a heterozygous state and occurred *de novo*. Most of the GRIN2B mutations that are associated with neurodevelopmental disorders have gain of function characteristics (Table 3).^49^ Clinically relevant rare variants of the NMDARs are enriched in a bi-lobed agonist binding domain (ABD) and pore-forming transmembrane domains (TMD) including three transmembrane helices (M1, M3, M4) and a reentrant loop (M2).^50^ Relevantly, some studies in healthy populations have demonstrated that different domains of *GRIN2B* do not exhibit a similar tolerance to missense variants, pinpointing those genetic variants in these sections are more likely to be pathogenic and function of that domains is critical.^51,52^ The most prevalent symptoms of affected individuals who harbored *GRIN2B *mutation were ID (94.9%), seizure (35.3%), DD (32.3%), and behavioral abnormality (30.3%). No prominent brain imaging finding was found in this group of patients (Table S2.7).

###  Diversity in Clinical Presentation

 It must be borne in mind that in channelopathies, seemingly, phenotypic heterogeneity even within family members with the same mutation does not solely emanate from variants in membrane ion channels and other unknown rare variants particularly in minor genes which change the effect of principal gene mutations are also involved.^26,32^ Also, the variable clinical spectrum may stem from the numerous isoforms created by alternatively spliced exons, and changing expression of distinct transcripts in tissue. For instance, in some individuals with *CACNA1C *mutation, the expression of mutation-bearing transcript is predominantly confined to the heart (exon 8A), while in others, there is a predominant neurologic expression (exon 8A).^36^ Within this context, mild to severe phenotype of the patients with the same mutation in the *SCN8A* (i.e., c.5630A > G, p.Asn1877Ser) has been attributed to mosaicism, protective genetic variants, or modifier mutations.^53^ Altogether, this evidence illustrates that in individuals with channelopathies, genotype-phenotype correlation is not straightforward as patients harboring the same mutation may have different clinical presentations. In other words, it would seem prudent to consider that there is an association between genotype and phenotype other than causality.

 In conclusion, regarding the critical role of brain ion channels in neuronal development, not surprisingly, mutations in genes encoding for these channels, both inherited and sporadic, alter membrane biophysical characteristics and are associated with neurodevelopmental disorders characterized by significant phenotypic and genetic heterogeneity. Our study provides further insight into the clinical and molecular features of disorders of ion channels and widens the clinical-genetic landscape of this group of disorders in patients with ID and/or DD; although we could not establish a definitive genotype-phenotype correlation. This highlights the fact that there is an association between the variants and diagnosed phenotypes.

## Supplementary Materials

Supplementary file 1 contains Figure S1.

Supplementary file 2 contains Tables S1.1 to S1.7.

Supplementary file 3 contains Tables S2.1 to S2.7.
