# Supplementary material for: Characterizing Genotypes and Phenotypes Associated with Dysfunction of Channel-Encoding Genes in a Cohort of Patients with Intellectual Disability
Source: Arch Iran Med. 2022 Dec 1;25(12):788–97. doi: 10.34172/aim.2022.124 (PMC10685845; doi:10.34172/aim.2022.124)
Supplement: Supplementary file 1 — contains Figure S1. [file aim-25-788-s001.pdf]

Arch Iran Med. December 2022;25(12):788-797
10.34172/aim.2022.124

Characterizing Genotypes and Phenotypes Associated with Dysfunction of Channel-Encoding Genes in a Cohort of Patients with Intellectual Disability

Naeim Ehtesham, PhD[1]; Meysam Mosallaei, PhD[1]; Maryam Beheshtian, MD, MPH, PhD[1]; Shahrouz Khoshbakht, PhD[1]; Mahsa Fadaee, MSc[2]; Raheleh Vazehan, MSc[2]; Mehrshid Faraji Zonooz, MSc[2]; Parvaneh Karimzadeh, MD[3]; Kimia Kahrizi, MD[1]; Hossein Najmabadi, PhD[1,2*]

[1]Genetics Research Center, University of Social Welfare and Rehabilitation Sciences, Tehran, Iran
[2]Kariminejad – Najmabadi Pathology & Genetics Center, Tehran, Iran
[3]Department of Pediatric Neurology, School of Medicine, Pediatric Neurology Research Center, Mofid Children's Hospital, Shahid Beheshti University of Medical Sciences, Tehran, Iran

(A-Family 1)

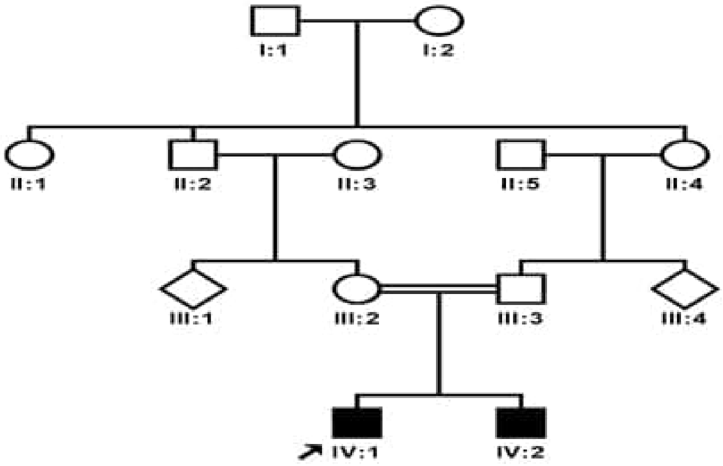

(B- Family 2)

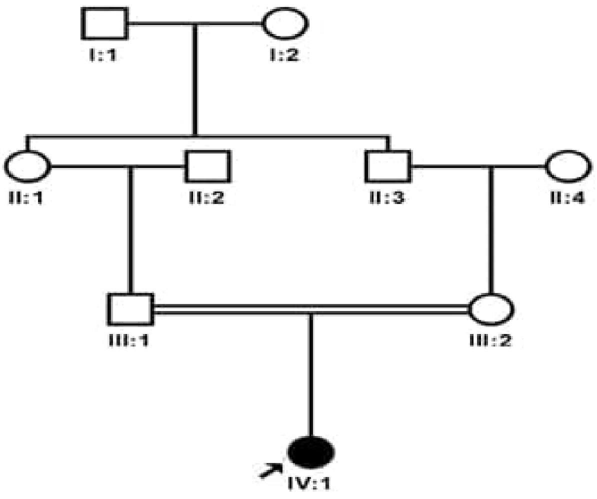

(C- Family 3)

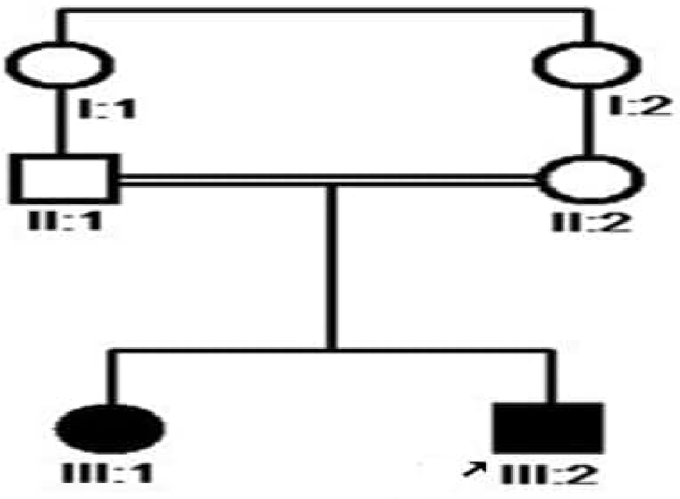

(D- Family 4)

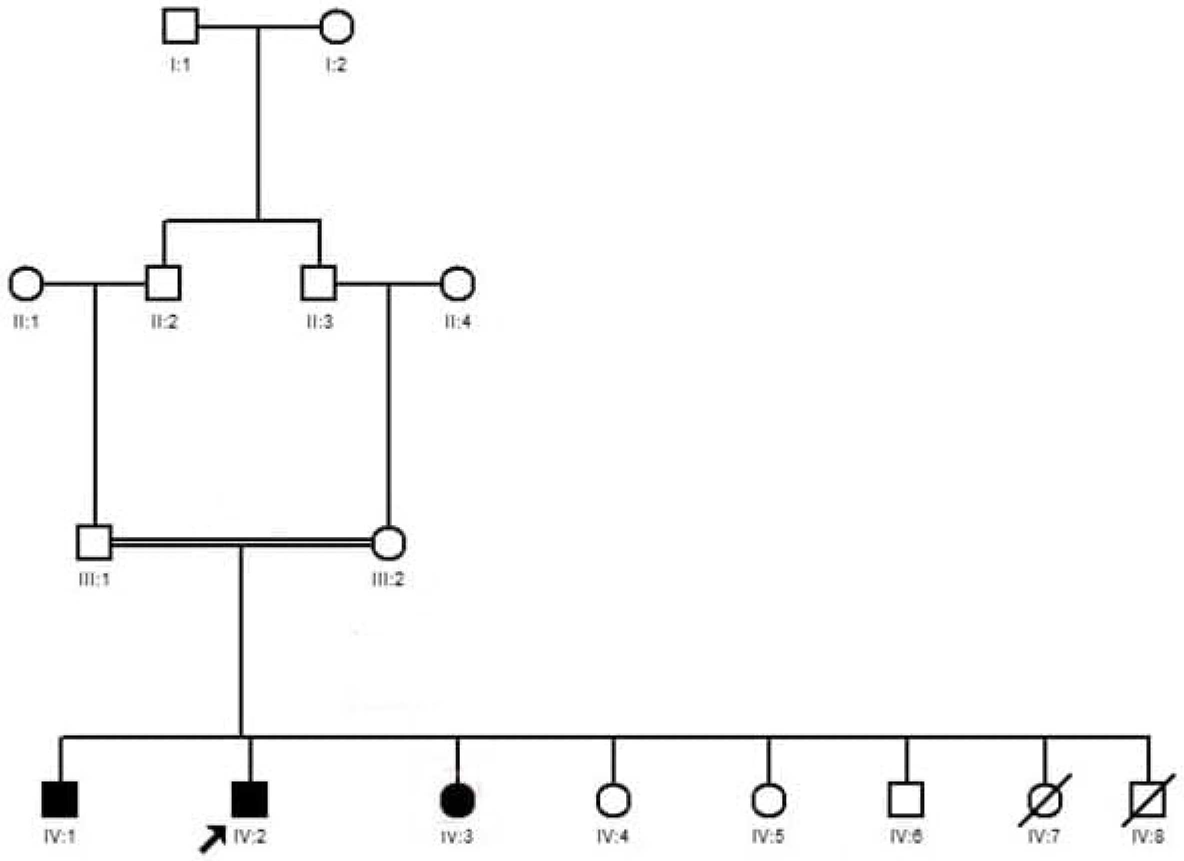

(E- Family 5)

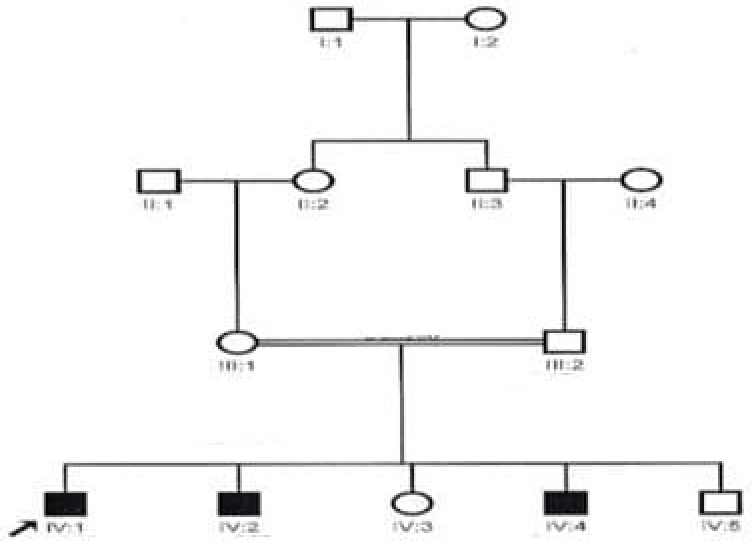

(F- Family 6)

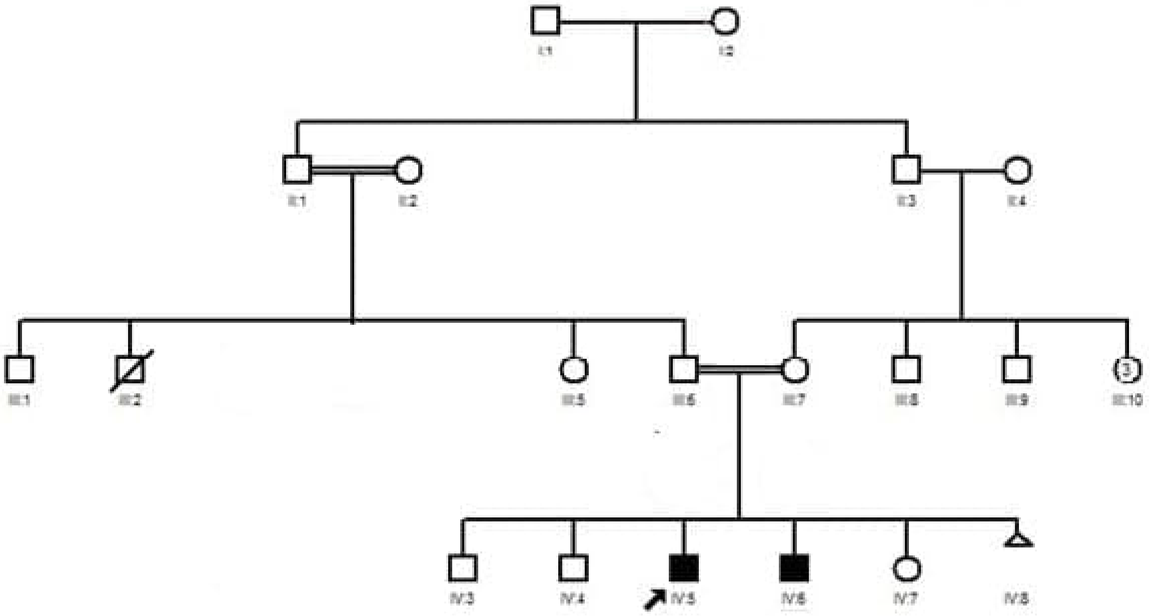

(G- Family 7)

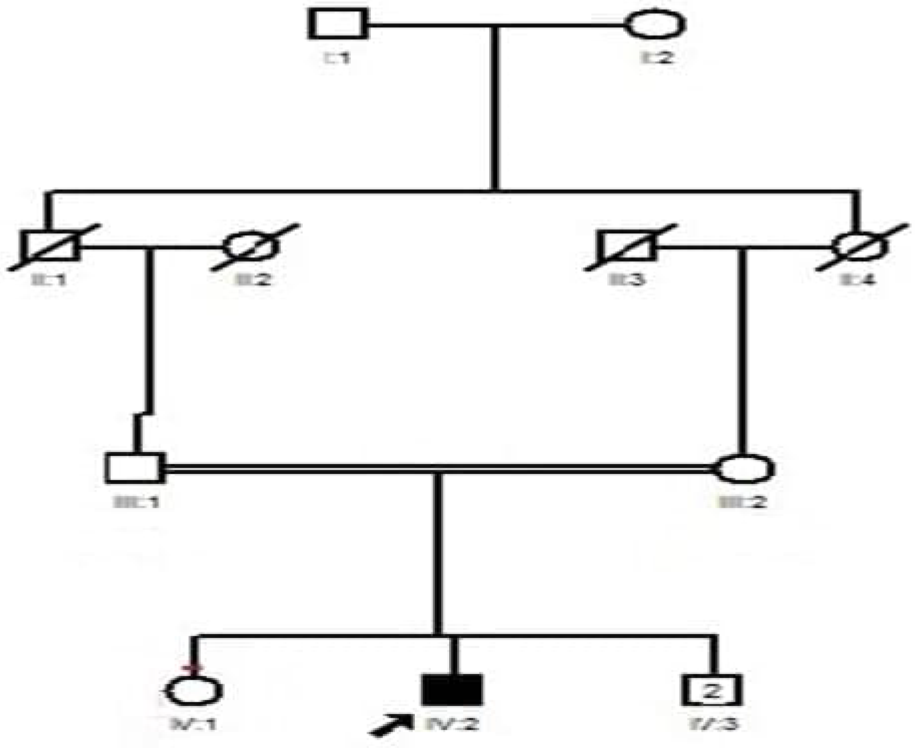

Figure S1. Pedigrees of 7 Iranian families with mutations in seven channel-encoding genes. A darkened circle or square represents an individual affected by the trait. An arrow on the lower-left corner of the individual indicates he/she is the proband. A: Pedigree of a consanguine family with 2 affected males with a variant in the *KCNJ10* gene. B: Pedigree of a consanguine family with 1 affected female with a variant in the *KCNQ3* gene. C: Pedigree of a consanguine family with 2 affected siblings (1 female and 1 male) with a variant in the *KCNK6* gene. D: Pedigree of a consanguine family with 3 affected individuals (2 males and 1 female) with a variant in the *CACNA1C* gene. E: Pedigree of a consanguine family with 3 affected male siblings with a variant in *CACNA1G* gene. F: Pedigree of a large and extended consanguine family with 2 affected male siblings with a variant in *SCN8A* gene. G: Pedigree of a consanguine family with a male proband with a variant in the *GRIN2B* gene.
